# Effect of trans-nasal humidified rapid insufflation ventilatory exchange on reflux and microaspiration in patients undergoing laparoscopic cholecystectomy during induction of general anesthesia: a randomized controlled trial

**DOI:** 10.3389/fmed.2023.1212646

**Published:** 2023-09-07

**Authors:** Yinyin Ding, Tianfeng Huang, Yali Ge, Ju Gao, Yang Zhang

**Affiliations:** Department of Anesthesiology, Northern Jiangsu People's Hospital, Yangzhou, Jiangsu, China

**Keywords:** trans-nasal humidified rapid insufflation ventilatory exchange, laparoscopic cholecystectomy, reflux, microaspiration, general anesthesia

## Abstract

**Background:**

Reflux aspiration is a rare but serious complication during induction of anesthesia. The primary aim of this study is to compare the incidence of reflux and microaspiration in patients undergoing laparoscopic cholecystectomy during induction of general anesthesia using either a facemask or trans-nasal humidified rapid insufflation ventilatory exchange.

**Methods:**

We conducted a single-center, randomized, controlled trial. Thirty patients were allocated to either a facemask or a trans-nasal humidified rapid insufflation ventilatory exchange (THRIVE) group. Pre-oxygenation for 5 min with a facemask or THRIVE, positive pressure ventilation for 2 min or THRIVE for 2 min after anesthesia induction was followed. Before endotracheal intubation, the secretion above and below the glottis was collected to measure pepsin content and analyze blood gas. The ELISA assay for supra- and subglottic human pepsin content was used to detect the presence of reflux and microaspiration. The primary outcome was the incidence of reflux and microaspiration. Secondary outcomes were apnea time, PaO_2_ before tracheal intubation, and the end-expiratory carbon dioxide partial pressure.

**Results:**

Patients in the THRIVE group had a significantly longer apnea time (379.55 ± 94.12 s) compared to patients in the facemask group (172.96 ± 58.87 s; *p* < 0.001). There were no differences observed in PaO_2_ between the groups. A significant difference in gastric insufflation, reflux, and microaspiration was observed between the groups. Gastric insufflation was 6.9% in the THRIVE group vs. 28.57% kPa in the facemask group (*p* = 0.041); reflux was 10.34% in the THRIVE group vs. 32.14% kPa in the facemask group (*p* = 0.044); and microaspiration was 0% in the THRIVE group vs. 17.86% kPa in the facemask group (*p* = 0.023).

**Conclusion:**

The application of THRIVE during induction of general anesthesia reduced the incidence of reflux and microaspiration while ensuring oxygenation and prolonged apnea time in laparoscopic cholecystectomy patients. THRIVE may be an optimal way to administer oxygen during the induction of general anesthesia in laparoscopic cholecystectomy patients.

**Clinical trial registration:**

Chinese Clinical Trial Registry, No: ChiCTR2100054086, https://www.chictr.org.cn/indexEN.html.

## Introduction

Trans-nasal humidified rapid insufflation ventilatory exchange (THRIVE), known as high-flow nasal cannula (HFNC) oxygen therapy, refers to a new type of oxygen therapy that directly delivers a specific concentration of high-flow air-oxygen mixed gas to the patient through heating and humidification without the need for a closed nasal catheter (1). THRIVE provides warmed and humidified gas at a maximum flow rate of 70 L/min, giving patients stable FiO_2_ while reducing anatomical dead space in the upper airway and increasing the patient’s intratracheal oxygen concentration (2). Studies have shown that continuous THRIVE can reduce the patient’s respiratory work while producing a degree of constant positive end-expiratory pressure (PEEP) effect (3–7). The positive pressure effect increases with increasing flow (8). For every 10 L/min increase in flow rate, the patient can obtain 1 cm H_2_O PEEP with a closed mouth and 0.5 cm H_2_O PEEP with an open mouth (9). The positive pressure effect produced by THRIVE opens the patient’s upper airway and reduces intrapulmonary shunts (10, 11), which further increases the patient^’^s oxygen reserve. The application of THRIVE during the induction of general anesthesia has been shown to prolong apnea time (10, 12–18). Pre-oxygenation with THRIVE can effectively reduce adverse events related to emergency endotracheal intubation in critically ill patients (16), and can ensure sufficient oxygenation induced by rapid anesthesia in emergency surgery patients (14).

Laparoscopic cholecystectomy (LC) is one of the most common elective abdominal operations (19). Patients with symptomatic gallbladder diseases have been reported to exhibit delayed gastric emptying despite following fasting guidelines. Gastric ultrasound assessment has revealed that 13% of patients scheduled for elective cholecystectomy had a full stomach because of symptomatic gallbladder disease (20). During the induction of anesthesia in general anesthesia patients, positive pressure-assisted ventilation is often accompanied by gastric insufflation, with an incidence of up to 50% (21). The increased intragastric pressure caused by a large amount of gastric insufflation, combined with the decreased tension of the lower esophageal sphincter and the suppression of the protective reflex of the upper airway under anesthesia, may further increase the risk of reflux aspiration in patients (13, 22). Anesthesiologists, therefore, remain vigilant against the high risk of reflux aspiration in patients during the induction period of general anesthesia.

Reflux aspiration is a phenomenon in which gastric acid, bile, and other gastric contents are abnormally regurgitated into the oropharynx and accidentally inhaled into the lungs. Reflux microaspiration is a rare but serious complication that occurs during the induction of anesthesia. How to effectively avoid reflux microaspiration caused by induction of general anesthesia has been a concern for anesthesiologists. Currently, the detection of pepsin can be considered a non-invasive method for effectively diagnosing reflux and microaspiration (23, 24). Still, there are no universally accepted standards for defining values of pepsin concentration for diagnosing reflux and microaspiration. For instance, Weitzendorfer et al. (25) showed a specificity of 86.2% and sensitivity of 41.5% for the diagnosis of reflux using a 216 ng/mL concentration of salivary pepsin as the threshold value. Similarly, Jaillette et al. (26) used a patient’s intratracheal pepsin concentration of 200 ng/mL as the diagnostic threshold for microaspiration.

Pre-oxygenation with THRIVE can ensure the patient’s full oxygenation without the need for a closed mask for positive pressure-assisted ventilation. This has potential value for anesthesia induction in people with a high risk of full stomach or reflux aspiration. However, it is unclear whether patients would benefit from the use of THRIVE during induction of anesthesia. We hypothesized that THRIVE could be beneficial to patients undergoing induction of general anesthesia for laparoscopic cholecystectomy. Therefore, we designed a randomized controlled study to observe the effect of THRIVE on reflux and microaspiration in laparoscopic cholecystectomy patients during induction of general anesthesia.

## Methods

### Study design

The study received approval from the Ethics Committee of Northern Jiangsu People’s Hospital (2021ky288), was registered in the China Clinical Trial Registration Center (ChiCTR2100054086), and was performed between 10 December 2021 and 31 March 2022. After obtaining written informed consent, adult patients (18–60 years old) who were scheduled to undergo elective laparoscopic cholecystectomy were recruited for the present study. Exclusion criteria were as follows: difficult airway; abnormal gastric anatomy; previous esophageal or gastric surgery; history of chronic obstructive pulmonary disease; history of gastroesophageal reflux; and history of nasal surgery. Patients were randomly allocated to either a trans-nasal humidified rapid insufflation ventilatory exchange or a standard facemask for pre-oxygenation. Allocation was completed using sealed envelopes assigned in a 1:1 ratio. The envelopes were numbered sequentially and were opened by the investigator after patient consent was obtained.

### Perioperative management

All patients routinely fasted before the operation (a minimum of 2 h for clear fluid and 8 h for solid intake). Standard peri-operative monitoring, including an electrocardiogram (ECG), non-invasive blood pressure (NIBP), and a pulse oximeter, was undertaken. An intravenous line was placed, and Ringer’s lactate solution was given peri-operatively. Ultrasonic images of the gastric antrum were collected in the supine position.

### Study protocol

Patients assigned to the facemask group were provided 100% oxygen for 5 min via a standard facemask, using a circle system with an oxygen rate of 6 L/min. The pressure mask was used to artificially assist positive pressure ventilation after the induction of anesthesia, the adjustable pressure-limiting (APL) valve was adjusted to 15 cmH_2_O, and then endotracheal intubation was performed at 2 min.

Patients in the THRIVE group were pre-oxygenated for 5 min using an Opti Flow-^™^ nasal high-flow cannula. The oxygen flow rate began at 30 L/min and increased to 50 L/min during anesthesia induction. The flow rate was immediately increased to 70 L/min after the patient’s consciousness completely disappeared. This flow was maintained until the tracheal tube was placed. The patient’s mouth was kept closed during the process, and endotracheal intubation was performed after 2 min. Chin lift and/or jaw thrust were used during apnea to maintain an open airway.

General anesthesia was induced using a titrated dose of 0.05 mg/kg midazolam, 0.4 μg/kg sufentanil, 2.0 mg/kg propofol, and was followed by 0.6 mg/kg rocuronium. Apnea time was defined as the period from the end of rocuronium injection until blood oxygen saturation (SpO_2_) decreased to 94%. Following the decrease of SpO_2_ to 94%, the ventilator was immediately connected for mechanical ventilation, and the end-expiratory carbon dioxide partial pressure (P_ET_CO_2_) during the first mechanical ventilation was recorded after intubation. Then, manual lung recruitment was performed until SpO_2_ returned to the level of entry. Heart rate and NIBP were maintained to fluctuate within the normal range during the operation, and vasoactive drugs were given when necessary.

Gastric antrum ultrasonography was performed in both groups before endotracheal intubation. The cross-sectional area (CSA) of the gastric antrum was measured, and the gastric insufflation was monitored. Before endotracheal intubation, the secretion above and below the glottis was collected to measure the content of pepsin to evaluate the occurrence of reflux and microaspiration. Blood gas analysis was performed before endotracheal intubation. The safe apnea time from the end of intravenous muscle relaxation to 94% reduction to SpO_2_ was recorded. Furthermore, P_ET_CO_2_ was recorded during the first mechanical ventilation after intubation.

The primary outcome was the incidence of reflux and microaspiration. Secondary outcomes were PaO_2_ before tracheal intubation, apnea time, and P_ET_CO_2_.

**Figure 1:**
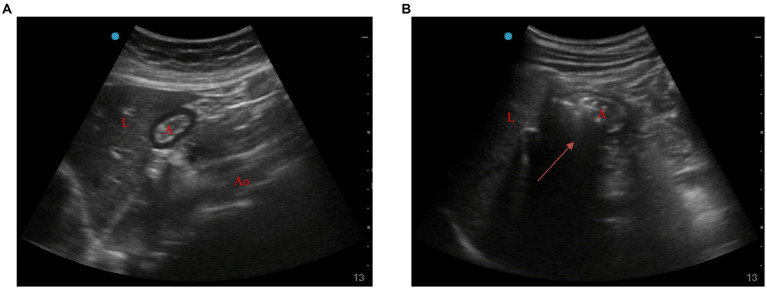
Ultrasonic images of gastric antrum before and after ventilation. **(A)** Before ventilation, **(B)** after ventilation (arrow). A, antrum; L, liver; Ao, aorta.

Ultrasonic examination of gastric antrum: A gastric ultrasound examination was performed using an ultrasound system with a 2–5 MHz convex array probe. The probe was placed along the sagittal plane of the epigastric area, and the gastric antrum was visualized in the parasagittal plane, just right of the midline, surrounded by the anterior left lobe of the liver and by the posterior pancreas. The CSA was measured by using a free tracing tool (27). During ultrasonic detection, gastric insufflation was determined by the significantly increased area in the sound shadow within the gastric antrum area or the typical “comet tail sign” (Figure 1) (28).

The enzyme-linked immunosorbent assay (ELISA) for supra- and subglottic human pepsin content in two groups of patients: After induction of anesthesia, the patient’s vocal canal was exposed using a visual laryngoscope. Under visual conditions, a single-use sampler was placed, the supra- and subglottic secretions were scraped, and the head of the sampler was broken off and collected into sterile EP tubes. The supra- and subglottic secretion specimens were lyophilized at −20°C for testing, and an ELISA kit (Shanghai Hepai Biotechnology Co., Ltd.) was used for the assay. The supra- and subglottic secretion was collected in a tube containing citric acid by centrifugation at 3,000 r/min for 10 min, and the supernatant was taken for the assay. The pepsin concentration of the sample was calculated according to the equation of the curve after the standard linear regression curve was drawn according to the concentration of the standard. A threshold value of 216 ng/mL of pepsin was defined for supraglottic secretion (25); a value of <216 ng/mL was defined as negative for reflux, and >216 ng/mL was defined as positive for reflux. A threshold value of 200 ng/mL was defined for subglottic secretions (26); a value of <200 ng/mL was defined as negative for microaspiration and >200 ng/mL was defined as microaspiration positive.

### Statistical analysis

The measurement data that was normally distributed was expressed as mean ± standard deviation ( $\bar{x}$ ±s). The measurement data that was not normally distributed was expressed by median (M) and interquartile interval (IQR). Numerical variables were analyzed using an independent samples *t*-test or the Mann–Whitney U test. Categorical data were analyzed using a chi-square test. A value of *p* < 0.05 was considered statistically significant. All tests were performed using SPSS Statistics26.

### Sample size estimation

No previous study has investigated the use of pepsin to define reflux microaspiration. To determine sample size, we randomly selected 20 patients according to the inclusion criteria in the study protocol for a preliminary trial, and these patients were not included in the formal study. Before anesthesia induction, 10 patients each received regular facemask oxygen inhalation and THRIVE pre-oxygenation, and the CSA of the gastric antrum was measured before intubation. The pre-tracheal intubation CSA areas of patients in the facemask and THRIVE groups were 3.58 ± 1.02 cm^2^ and 3.06 ± 0.98 cm^2^, respectively.

Using a type-1 error of 5% and type-2 error of 20% (power 80%), a sample size of 25 patients in each group was calculated. However, to account for attrition and study dropouts due to equipment errors (i.e., the replacement of the analysis package of the blood gas analyzer), we included 60 total patients, and the sample size was increased to 30 per group.

## Results

### Patient characteristics

**Figure 2:**
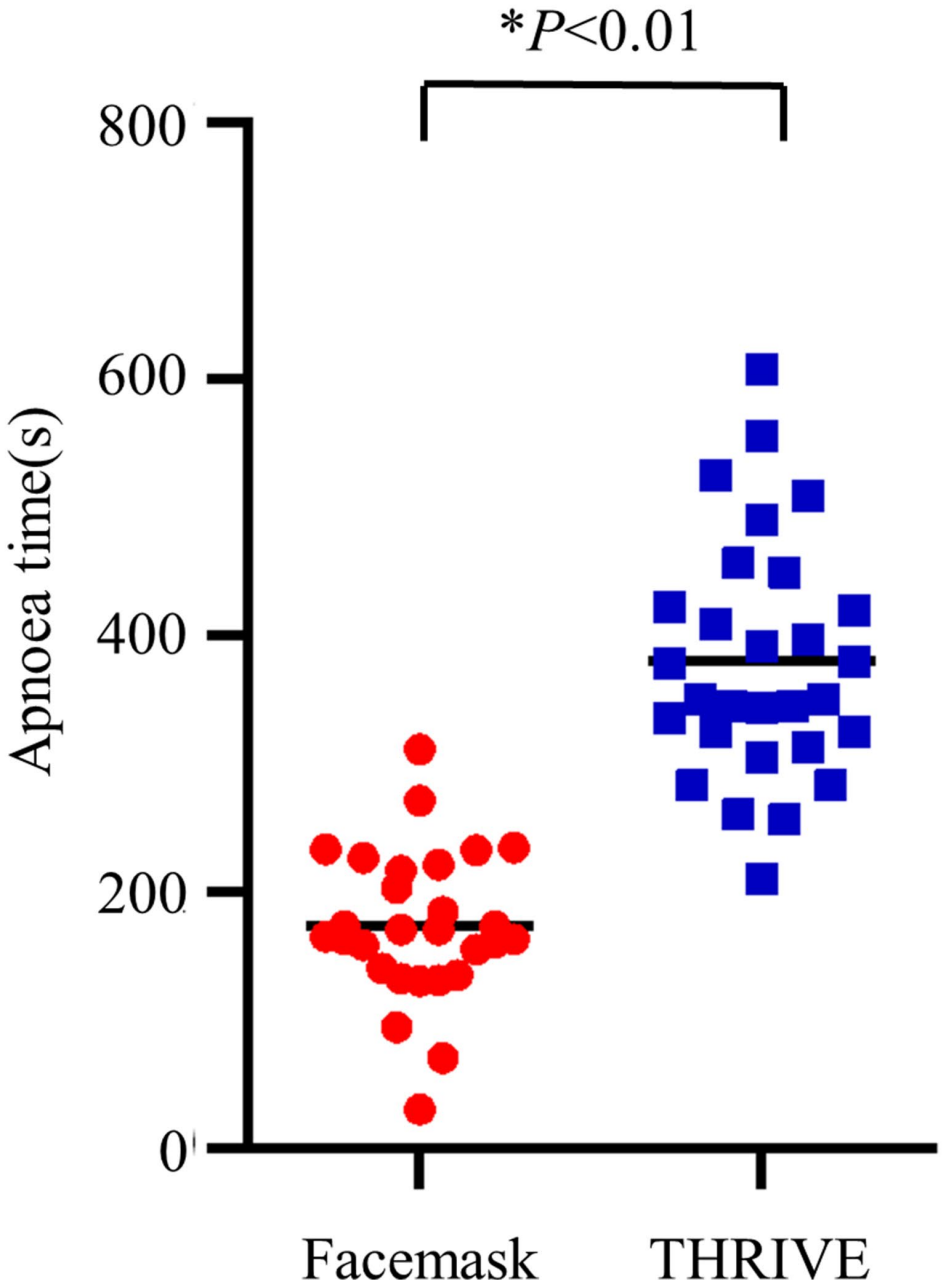
Apnea time. THRIVE pre-oxygenation (blue squares); facemask pre-oxygenation (red dots).

Seventy patients were assessed for eligibility. Ten patients were excluded (Figure 2), and 60 patients were included. In one patient, the ultrasonographic examination of gastric antrum CSA was not successful due to interference of intestinal flatulence; one patient failed to complete the blood gas analysis on time due to the replacement of the blood gas analyzer; and one patient failed to keep their mouth closed per requirement. Participant characteristics are summarized in Table 1.

**Table 1:** Characteristics of 57 patients pre-oxygenated with THRIVE or facemask for induction of anesthesia.

	Facemask (*n* = 28)	THRIVE (*n* = 29)	*p*-value
Age: years	53 (12)	52 (16)	0.637
Gender: male/female	13/15	12/17	0.701
Height: m	1.7 ± 0.1	1.6 ± 0.1	0.352
Weight: kg	69 ± 12	62 ± 10	0.089
ASA physical status: I/II	13/15	15/14	0.689
BMI: kg/m^2^	24 ± 2	23 ± 2	0.084
Hypertension	6 (21.4%)	5 (17.2%)	0.689
Diabetes	2 (7.1%)	2 (6.9%)	1.000
Baseline HR: beats/min	81 ± 14	78 ± 12	0.357
Baseline MAP: mmHg	104 ± 13	96 ± 12	0.053
Baseline SpO_2_: %	98 (1)	99 (2)	0.535
**Preoperative diagnosis**			
Gallstone	25 (89.3%)	26 (89.7%)	
Adenomyosis of gallbladder	2 (7.1%)	1 (3.4%)	
Gallbladder polyps	1 (3.6%)	2 (6.9%)	
Anesthesia time: min	55 (34)	50 (23)	0.268
Operation time: min	40 (30)	35 (18)	0.094
Fluid infusion: mL	1,000 (0)	1,000 (0)	0.531
Hemorrhage: mL	5 (5.0)	5 (2.5)	0.716

Values are mean (SD), median (IQR), or number (proportion).

ASA, American Society of Anesthesiologists; BMI, body mass index; HR, heart rate; MAP, mean arterial pressure; SpO_2_, blood oxygen saturation.

### Apnea time, arterial blood gas, and the first breath after intubation

**Figure 3:**
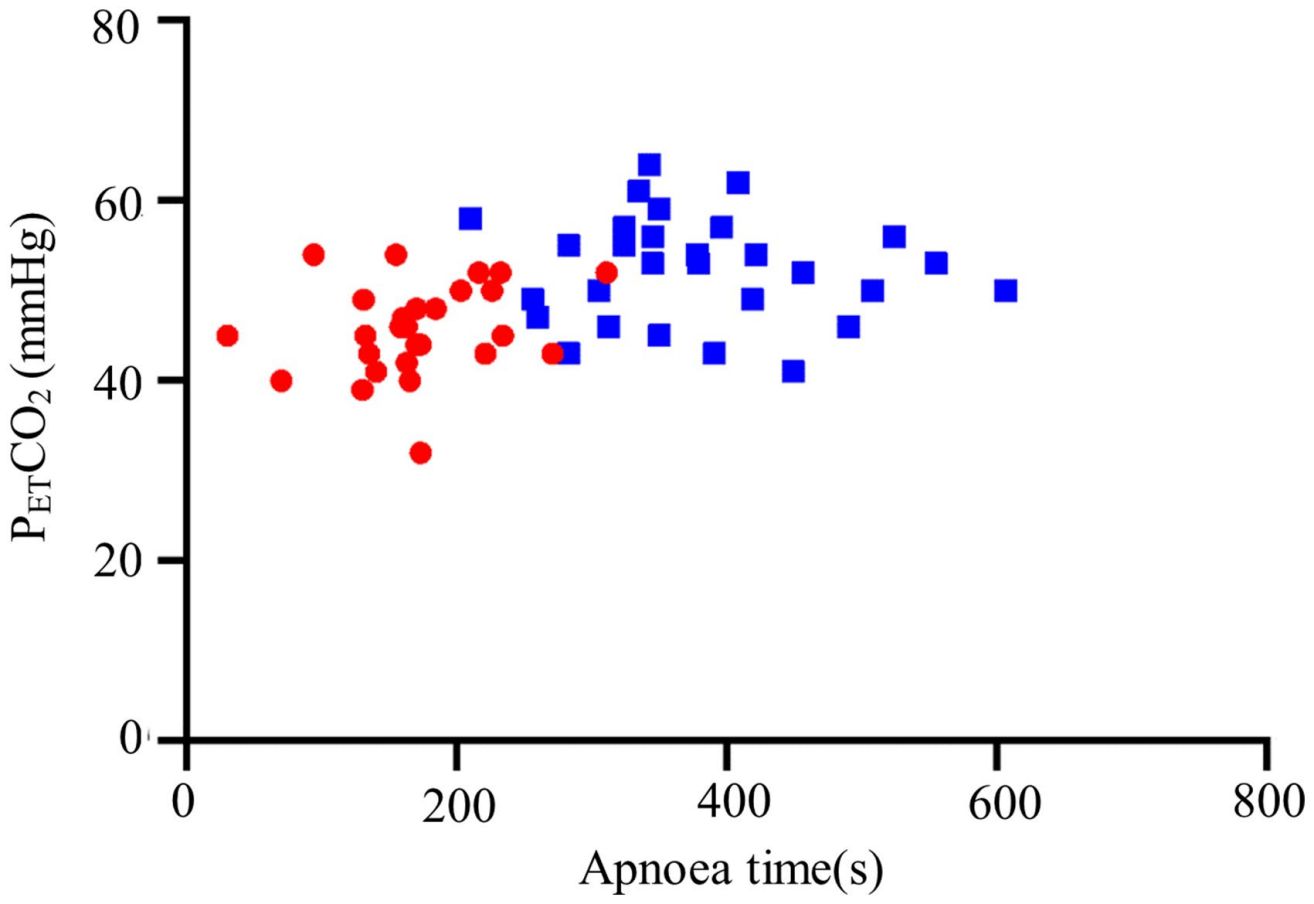
P_ET_CO₂ during the first breath after intubation and apnea time. THRIVE (blue squares); facemask (red dots).

We observed a longer apnea time in the THRIVE group compared to the facemask group (173 ± 59 s vs. 380 ± 94 s, *p* < 0.001) (Table 2). Increased levels of P_ET_CO₂ in and a higher level of PaO_2_ before intubation were seen in the THRIVE group (Table 2). Other conditions were similar between the facemask and THRIVE groups (Table 2; Figures 2, 3).

**Table 2:** P_ET_CO₂ in first breath after intubation, blood gas analysis, and apnea time.

	Facemask (*n* = 28)	THRIVE (*n* = 29)	*p*-value
PaO_2_: mmHg	192 ± 119	347 ± 131	0.000
PaCO_2_: mmHg	54 ± 6	54 ± 8	0.129
pH	7.3 ± 0.4	7.3 ± 0.4	0.719
Lac: mmol/L	0.6 (0.3)	0.6 (0.3)	0.570
Hct: %	37 ± 5	37 ± 5	0.915
HCO_3_^−^: mmol/L	27.1 ± 1.9	28.4 ± 2.9	0.054
Hb: g/dL	11.4 ± 2.5	11.5 ± 1.7	0.848
Apnea time: s	173 ± 59	380 ± 94	0.000
P_ET_CO₂ in first breath after intubation; mmHg	46 ± 5	52 ± 6	0.000

Values are mean (SD) or median (IQR).

### CSA, gastric insufflation, reflux, and microaspiration

The baseline CSA and CSA before intubation did not differ between the facemask and THRIVE groups, but statistically significant reductions in gastric insufflation, reflux, and microaspiration were observed in the THRIVE group (Table 3). Five patients (17.86%) in the group that was pre-oxygenated with a facemask had microaspiration. No microaspiration was seen in the THRIVE group (*p* = 0.023; Table 3).

**Table 3:** CSA, gastric insufflation, reflux, and microaspiration.

	Facemask (*n* = 28)	THRIVE (*n* = 29)	*p*-value
Baseline CSA; cm^2^	3.1 ± 1.1	3.0 ± 0.9	0.780
CSA>3.4 cm^2^	9 (32.1%)	6 (20.7%)	0.326
CSA before intubation; cm^2^	3.7 ± 1.0	3.2 ± 1.0	0.046
CSA>3.4 cm^2^	19 (67.9%)	9 (31.0%)	0.005
Gastric insufflation	8 (28.6%)	2 (6.9%)	0.041
Reflux	9 (32.1%)	3 (10.3%)	0.044
Micro aspiration	5 (17.9%)	0 (0%)	0.023

Reflux was defined as supraglottic pepsin concentration > 216 ng/mL, and microaspiration was defined as subglottic pepsin concentration > 200 ng/mL.

## Discussion

Our main result was that the use of THRIVE during induction of anesthesia reduced the incidence of regurgitation and microaspiration in patients. This may be because the THRIVE oxygen delivery method can effectively circumvent gastric inlet caused by artificial positive pressure-assisted ventilation. Routine mask-assisted positive pressure ventilation during induction of anesthesia is frequently complicated by gastric insufflation (21). This can cause gastric dilatation, leading to increased intragastric pressure, which can increase the risk of regurgitant aspiration in patients (13, 22). In the present study, the incidence of gastric insufflation significantly decreased in patients in the THRIVE group compared to the group on conventional mask ventilation. A previous study demonstrated that a maximum inspiratory airway pressure of <15 cm H_2_O allowed adequate pulmonary ventilation with reduced gastric inlet (29), whereas THRIVE produced a stable and continuous positive intra-airway pressure effect without mask positive pressure ventilation (9).

For every 10 L/min increase in flow rate, the patient can obtain 1 cm H_2_O PEEP with a closed mouth and 0.5 cm H_2_O PEEP with an open mouth (9), which results in a PEEP of less than 10 cm H_2_O even at oxygen flows up to 70 L/min (8, 30). In our study, pepsin concentrations were used to define reflux and microaspiration. However, a previous study by Sjöblom et al. (14) reported that the person intubating was responsible for checking the pharynx for signs of reflux, and thus, this measurement may be more informative compared to assessing pepsin concentrations.

Ultrasound measurement of the patient’s CSA was used in the present study to further assess the effect of THRIVE on the patient’s gastric contents. A total of 91% sensitivity and 71% specificity for the diagnosis of a full stomach was obtained at a threshold value of 3.4 cm^2^ of CSA (31). These results showed that THRIVE reduced the patient’s pre-tracheal intubation CSA and decreased the proportion of patients with CSA >3.4 cm^2^. Moreover, THRIVE intervention did not affect the basal value of the patient’s gastric contents. Rather, it reduced the occurrence of gastric inlet and failed to cause an increase in gastric contents compared to traditional induction of anesthesia with positive pressure-assisted ventilation by mask. THRIVE can reduce the occurrence of regurgitation and microaspiration during induction of anesthesia in LC patients. McLellan et al. (21) performed an ultrasound assessment of 1:1 gastric contents before and after HFNC intervention in 60 healthy adult volunteers who fasted for at least 8 h. The results showed no signs of gastric distension or increase in gastric secretions when using HFNC during spontaneous ventilation in healthy adult volunteers.

In the present study, PaCO_2_ in arterial blood before tracheal intubation was not significantly different between the facemask and THRICE patients, which may be related to the fact that during apnea, THRIVE promotes the clearance of CO_2_ from the anatomical dead space and reduces the repetitive inhalation of CO_2_, achieving partial clearance of CO_2_ (10, 17, 32). The results of our study showed that the use of THRIVE during induction of anesthesia at the same time as oxygen administration significantly prolonged the time to apnea. In addition, we demonstrated higher P_ET_O₂ levels in the THRIVE patient group at the first breath after intubation. These results are inconsistent with previous studies (14, 17). Apneic oxygenation using THRIVE has been shown to generate a slower increase in arterial CO_2_ over time (10). This finding may be related to the longer safe apnea time, with slow transient CO_2_ accumulation inevitably occurring as the patient’s apnea time prolongs. Similar studies have shown that oxygen administration with THRIVE significantly prolongs the time to 95% SpO_2_ in obese patients compared to regular mask oxygen administration (18). However, prolonged (30 min) apneic oxygenation with THRIVE can be limited by hypercapnia and severe acidosis (33), suggesting that hypercapnia may be a limitation of the application of THRIVE during apnea.

There are a number of limitations to our study. First, the study could not be blinded due to the nature of the intervention. Second, the study population consisted of mostly young and healthy patients. The value of THRIVE in the induction period of general anesthesia for additional populations, such as patients with poor preoperative pulmonary function and older adult and obese patients, needs further investigation. Finally, the use of THRIVE with differing oxygen flow rates and the duration of oxygen inhalation during the induction of anesthesia should be explored.

In conclusion, the application of THRIVE during the induction of general anesthesia can ensure patient oxygenation, prolong apnea time, and reduce the occurrence of reflux and microaspiration in laparoscopic cholecystectomy patients. Thus, THRIVE could improve oxygen administration during the induction of general anesthesia in laparoscopic cholecystectomy patients.

## Data availability statement

The datasets presented in this study can be found in online repositories. The names of the repository/repositories and accession number(s) can be found in the article/Supplementary material.

## Ethics statement

The studies involving humans were approved by Ethics Committee of Northern Jiangsu People’s Hospital (2021ky288), was registered in China Clinical Trial Registration Center (ChiCTR2100054086). The studies were conducted in accordance with the local legislation and institutional requirements. The participants provided their written informed consent to participate in this study.

## Author contributions

YD: writing original draft. TH and YZ: data analysis. YG and YZ: writing review and editing. JG: supervision and conceptualization. All authors contributed to the article and approved the submitted version.

## Funding

This research was supported by The National Natural Science Fund, China (82172190, 82101299).

## Conflict of interest

The authors declare that the research was conducted in the absence of any commercial or financial relationships that could be construed as a potential conflict of interest.

## Publisher’s note

All claims expressed in this article are solely those of the authors and do not necessarily represent those of their affiliated organizations, or those of the publisher, the editors and the reviewers. Any product that may be evaluated in this article, or claim that may be made by its manufacturer, is not guaranteed or endorsed by the publisher.

## Supplementary material

The Supplementary material for this article can be found online at: https://www.frontiersin.org/articles/10.3389/fmed.2023.1212646/full#supplementary-material
