# Supplementary material for: Effect of trans-nasal humidified rapid insufflation ventilatory exchange on reflux and microaspiration in patients undergoing laparoscopic cholecystectomy during induction of general anesthesia: a randomized controlled trial
Source: Front Med (Lausanne). 2023 Sep 7;10:1212646. doi: 10.3389/fmed.2023.1212646 (PMC10512709; doi:10.3389/fmed.2023.1212646)
Supplement: Supplementary file 1 [file Data_Sheet_1.doc]

**Allocation**

**Analysis**

**Enrollment**

Assessed for eligibility (n= 70)

Excluded (n= 10)

♦  Not meeting inclusion criteria (n=5)

♦  Declined to participate (n=3)

♦  Other reasons (n=2)

Analysed (n=28)
♦ Excluded from analysis (Excluded from analysis (the interference of peripheral intestinal flatulence; failed to complete the blood gas analysis) (n = 2)

Allocated to pre-oxygenation with facemask (n=30)

♦ Received allocated intervention (n=30)

♦ Did not receive allocated intervention (n=0)

Analysed (n=29)
♦ Excluded from analysis (not keep the mouth closed as required) (n=1)

Allocated to pre-oxygenation with THRIVE (n=30)

♦ Received allocated intervention (n=30)

♦ Did not receive allocated intervention (n=0)

Randomized (n=60)
